# Determination of hepatitis B, C and D prevalence among urban and Amerindian populations from the Eastern Brazilian Amazon: a cross sectional study

**DOI:** 10.1186/s12879-018-3279-2

**Published:** 2018-08-20

**Authors:** Livia Melo Villar, Flavio Augusto Pádua Milagres, Elisabeth Lampe, Helena Medina Cruz, Leticia de Paula Scalioni, Monica de Avelar Figueiredo Mafra Magalhães, Anselmo Rocha Romão, Renata Gracie, Vanessa Salete de Paula

**Affiliations:** 1 Laboratory of Viral Hepatitis, Oswaldo Cruz Institute, FIOCRUZ, Helio and Peggy Pereira Pavillion – Ground Floor - Room B09, FIOCRUZ Av. Brasil, 4365 - Manguinhos –, Rio de Janeiro, RJ 210360-040 Brazil; 2 Federal University of Tocantins, Tocantins, Brazil; 3 Laboratory of Information in Health, Institute of Communication and technological and scientific information in Health (ICICT), FIOCRUZ, Rio de Janeiro, Brazil; 4 Molecular Virology Laboratory, Oswaldo Cruz Institute, FIOCRUZ, Rio de Janeiro, Brazil

**Keywords:** Hepatitis B, Hepatitis C, Hepatitis D, Prevalence, Amerindians, Eastern Amazon, North Brazil

## Abstract

**Background:**

This study was conducted to determine the prevalence of HBV, HCV, and HDV in urban populations and Amerindians living in the state of Tocantins (Eastern Amazon).

**Methods:**

A total of 948 individuals were recruited in Tocantinopolis city (Tocantins state) of whom 603 were Amerindians (from 6 tribes) and 345 were non-Amerindians (6 urban areas of Tocantinópolis city). Anti-HCV, HBsAg, anti-HBc, anti-HBs, anti-HBc IgM, anti-HBe, HBeAg, and anti-delta antibodies were determined using enzyme immunoassay.

**Results:**

HBV cleared infection (both anti-HBc/anti-HBs+), chronic inactive/immune controlled HBV infection (anti-HBc + only), previous HBV vaccination (anti-HBs + only), active HBV infection (HBsAg+), individuals susceptible to HBV, and anti-HCV reactivity were found in 12.9, 1.8, 27.2, 0.5, 57.7, 1.2% in Amerindians and 12.1, 2.0, 37.1, 0.3, 55.4, 0.3% in non-Amerindians respectively. Out of 139 anti-HBc reactive individuals, 70 were anti-HBe reactive and none presented HBeAg or anti-HBc IgM. Anti-HBc prevalence was associated to older age (*p* < 0.0001). Overall anti-Delta prevalence was 0.3% and regarding anti-HBc reactive individuals, anti-delta prevalence was 3.4 and 0% in Amerindians and non-Amerindians respectively.

**Conclusions:**

Overall low prevalence of HBV and HCV infection was found in the populations studied, but high HBV and HCV prevalence was observed in Amerindians compared to non-Amerindians suggesting that these individuals have a higher likelihood of acquiring to these infections. Anti-delta antibodies were found among Amerindians from Eastern Amazon suggesting a risk for this population. Of note is that nearly half of Amerindians had no anti-HBs, indicating a need for HBV vaccination campaigns in this population.

## Background

Hepatitis B, C and D viruses, transmitted predominantly through parenteral routes, represent a considerable threat to public health. Worldwide, approximately 257 million people are chronically infected with hepatitis B virus (HBV), 71 million with chronic hepatitis C virus (HCV) and 15 million with HBV and hepatitis D virus (HDV) [1–3].

Multicenter studies conducted in several regions of Brazil have demonstrated seroprevalence rates of anti-HBc (HBV past exposure) of 11.6% and anti-HCV of 3.22% in individuals aged 10 to 69 years old from North region of Brazil [4, 5]. In the period spanning 1999 to 2016, approximately 561,000 confirmed cases of viral hepatitis were reported to health authorities in Brazil. Of these, 212.031 (37,8%) hepatitis B, 182.389 (32,5%) hepatitis C and 3.791 (0,7%) hepatitis D cases were identified [6].

HBV, HCV and HDV prevalence varies considerably according to geographical region of the country, where the highest HCV prevalence (3.22%) is observed in the North region [5]. The highest rates of HBV carriers and more than 70% of reported HDV cases are also observed in the Amazon Region located in the North of the country [6, 7]. HDV infection is also common in acute hepatitis B cases (29%) and fulminant hepatitis cases (74%) in the North of Brazil [8].

Some specific groups, like Amerindians, people who use drugs, beauty professionals, and military personnel are at higher risk of viral hepatitis acquisition [7, 9–12]. In 2010 the Brazilian census estimated the population of Amerindians at 896,917 individuals, corresponding to 0.47% of the general population with nearly half of them living in the Northern region of the country [13]. HBsAg prevalence varies from 0 to 20.6%, Anti-HDV from 0 to 7.7% [7] and anti-HCV from 1.4 to 1.6% [14] in the Northern Amerindian population.

Viral hepatitis is higher in the Northern areas where many Amerindians live, and even within this region prevalence varies between different areas. Most prevalence studies of viral hepatitis from the North of Brazil were conducted in Western Amazon [15–20] and there is a gap in the literature in terms of geographical variations. As a result, this information is needed to develop targeted screening programs.

This study was conducted to estimate the prevalence rates of HBV, HCV and HDV markers between Amerindians and the general population residing in the state of Tocantins (Eastern Amazon) located in the Northern region of Brazil.

## Methods

A cross-sectional survey regarding hepatitis B, C and D prevalence was conducted using a non-probability sampling method among Amerindians and non-Amerindians living in Eastern Amazon. Consecutive sampling was used in which every subject meeting the criteria of inclusion is selected until the required sample size is achieved in this setting. The North Region of Brazil is comprised of seven States (Acre, Amapá, Amazonas, Pará, Rondônia, Roraima and Tocantins) that, together with Mato Grosso and Maranhão States form the Brazilian Amazon. The West Amazon region comprises the states of Amazonas, Acre, Rondônia and Roraima. The East Amazon is comprised of the states of Maranhão, Pará, Amapá, Mato Grosso and Tocantins. The study was carried out from June 2011 to June 2017 and samples were collected from 6 tribes of Amerindians and 6 urban areas of Tocantinópolis city in June 2011.

Eligibility criteria for participation of this study were: residence in the area and the provision of informed consent. Exclusion criteria were: confusion at the time of recruitment and disagreement with the terms of the informed consent.

### Blood sampling and viral hepatitis detection

Each participant donated a blood sample (5 mL) by venipuncture using a vacutainer device. The sample was allowed to clot to separate the serum for analysis and was stored at − 20 °C until analysis.

Serum samples were tested for HBsAg, anti-HBc, anti-HBs, anti-HCV, using commercial enzyme immunoassay (ELISA) kits (Diasorin, Pomezia, Italy), according to the manufacturer’s guidelines. HBsAg or Anti-HBc reactive samples were also assayed for anti-HBc IgM and anti-delta antibodies using ELISA kit (Diasorin), and anti-HBe and HBeAg using eletrochemiluminescence assay (ECLIA) (Roche, USA).

Samples found to be negative on preliminary screening were considered seronegative. Samples that initially tested borderline or positive were retested using ELISA to confirm the results. Indeterminate samples were excluded from the analysis.

Anti-HCV reactive samples were submitted to real time PCR (Cobas Taqman HCV 2.0, Roche, USA), which has a dynamic range of linear quantification of 20 to 1.7 × 10^8^ IU/mL. HBsAg-reactive samples were submitted to real time PCR (Cobas Taqman HBV Test, Roche, USA), which has a dynamic range of linear quantification of 29 to 1.1 × 10^8^ IU/mL.

### Data collection and analysis

Age, gender and residential location data were obtained from each participant and entered along with serological results into (Microsoft Excel) data spreadsheets.

Prevalence was calculated for HBV, HCV and HDV markers in the studied population. Descriptive statistics were generated for the data. Chi square test for independence and exact Fisher’s tests were used to compare categorical variables according to anti-HBc status. A *p*-value < 0.05 was considered statistically significant. All calculations were performed using the Statistical Package for the Social Sciences (SPSS for Windows, release 20.0; SPSS, Chicago, IL, USA).

The spatial representation of HBV markers analyzed was based on cartography of the digital mesh of the 2010 census, [13] and data of HBV markers according localities of the municipality of Tocantinópolis in the state of Tocantins. For the adaptation of the analyzed locations to the census tracts, the National Register of Addresses for Statistical Purposes of the IBGE was used [13]. Thematic maps of HBV markers were created using the Geographic Information System (GIS) ArcGis version 10.4.

### Ethical consideration

The study project was reviewed and approved by Fiocruz ethics Committee and Brazilian National Ethics Council, Brazilian Indian Foundation and local tribe leaders. For this study, authorization was solicited and obtained from the Indians’ authorities at the national, regional, and local levels.

Respondents were assured about confidentiality, that their participation was voluntary and that they had full rights to withdraw from the study at any time. All participants were given a verbal explanation of the objectives and methodology of the research and were included in the study only after obtaining written signed informed consent. Parent or guardian gave written signed informed consent on behalf of any participants under the age of 18. All individuals who tested positive were sent to public health clinics to receive treatment.

## Results

In the present study, 948 individuals were included aging 0 to 90 years, 569 were Amerindians and 379 were non-Amerindians. Most of them were females (53.3%) and mean age of individuals was 27.8 ± 20.6 years. The socio-demographic characteristics of the 948 individuals included in this study are shown in Table 1.

**Table 1:** Hepatitis B and C virus markers among individuals from Amerindian tribes and urban areas of Tocantinopolis city (*n* = 948)

	Number Tested	HBsAg - anti-HBc + anti-HBs + (HBV cleared infection)	HBsAg - anti-HBc + anti-HBs - (Chronic inactive/immune controlled HBV infection)	HBsAg - anti-HBc – anti-HBs + (HBV Vaccination group)	HBsAg + anti-HBs – (HBV active infection)	HBsAg – anti-HBc - anti-HBs – (HBV susceptible individuals)	anti-HCV +
Total population studied	948 (100%)	120 (12.6%)	18 (1.9%)	268 (28.3%)	4 (0.4%)	538 (56.7%)	8 (0.8%)
Sex							
Female	505 (53.3%)	56 (11.1%)	9 (1.8%)	143 (28.3%)	2 (0.4%)	296 (58.6%)	1 (0.2%)
Male	443 (46.7%)	64 (14.4%)	9 (2.0%)	125 (28.2%)	2 (0.4%)	242 (54.6%)	7 (1.6%)
Age group (years)							
0–20	438 (46.2%)	9 (2.0%)	3 (0.7%)	166 (0.4%)	2 (0.4%)	258 (58.9%)	0 (0.0%)
21–40	277 (29.2%)	31 (11.2%)	0 (0.0%)	88 (31.7%)	1 (0.4%)	157 (56.6%)	4 (1.4%)
41–60	142 (14.9%)	49 (34.5%)	5 (3.5%)	9 (6.3%)	1 (0.7%)	78 (54.9%)	3 (2.1%)
> 60	91 (9.6%)	31 (34.1%)	10 (11.0%)	5 (5.5%)	0 (0.0%)	45 (49.4%)	1 (1.1%)
Location							
Amerindian tribes	603 (63.6%)	78 (12.9%)	11 (1.8%)	164 (27.2%)	3 (0.5%)	348 (57.7%)	7 (1.2%)
Prata village	53 (5.6%)	2 (3.8%)	0 (0.0%)	24 (45.3%)	0 (0.0%)	27 (50.9%)	0 (0.0%)
Girassol village	89 (9.4%)	16 (17.9%)	0 (0.0%)	14 (15.7%)	1 (1.1%)	58 (65.2%)	1 (1.1%)
Mariazinha village	206 (21.7%)	29 (14.1%)	1 (0.5%)	51 (24.7%)	1 (0.5%)	124 (60.2%)	3 (1.4%)
Riachinho village	18 (1.9%)	3 (16.6%)	0 (0.0%)	6 (33.3%)	0 (0.0%)	9 (50.0%)	0 (0.0%)
Serrinha village	66 (7.0%)	1 (1.5%)	0 (0.0%)	17 (25.7%)	0 (0.0%)	48 (72.7%)	1 (1.5%)
Folha Grossa village	171 (18.0%)	27 (15.8%)	10 (5.8%)	52 (30.4%)	1 (0.6%)	82 (0.5%)	2 (1.2%)
Non-Amerindian (Urban areas)	345 (36.4%)	42 (12.2%)	7 (2.0%)	128 (37.1%)	1 (0.3%)	191 (55.4%)	1 (0.3%)
Cacau urban area	60 (6.3%’)	3 (5.0%)	1 (1.7%)	12 (20.0%)	0 (0.0%)	44 (73.3%)	1 (1.7%)
Mumbuco urban area	81 (8.5%)	9 (11.1%)	1 (1.2%)	32 (39.5%)	0 (0.0%)	39 (48.1%)	0 (0.0%)
BelaVista urban area	10 (1.0%)	2 (20.0%)	0 (0.0%)	2 (20.0%)	0 (0.0%)	6 (60.0%)	0 (0.0%)
Urban area block 18	84 (8.9%)	10 (11.2%)	3 (3.6%)	25 (29.7%)	1 (1.2%)	45 (53.6%)	0 (0.0%)
Urban area block 22	61 (6.4%)	5 (8.2%)	2 (3.3%)	21 (34.4%)	0 (0.0%)	33 (54.1%)	0 (0.0%)
Tocantinópolis Downtown	49 (5.2%)	13 (26.5%)	0 (0.0%)	36 (73.5%)	0 (0.0%)	24 (49.0%)	0 (0.0%)

Markers of HBsAg, anti-HBc and anti-HBs were detected in 4, 138, and 388 individuals giving an overall prevalence of 0.4, 14.5, 40.9%, respectively. Among the 4 HBsAg reactive individuals, none of them presented HBeAg or anti-HBc IgM and only one presented detectable anti-HBc, anti-HBe and HBV DNA (viral load of 6469 IU/mL). Of the 138 anti-HBc-reactive individuals, 70 were anti-HBe-reactive and none presented HBeAg or anti-HBc IgM. Anti-HBc prevalence was positively associated with older age (*p* < 0.0001) in bivariate analyses (Table 2).

**Table 2:** Bivariate analysis of demographic factors associated to anti-HBc prevalence in the population studied

Variable	Total Anti-HBc		Bivariate analysis *P* Value
	Reactive *n* = 138 (%)	Non reactive *n* = 810 (%)	
Age (years)			
0–20	12 (8.7)	426 (52.6)	< 0.0001
21–40	31 (22.5)	246 (30.4)	
41–60	54 (39.1)	88 (10.9)	
> 60	41 (29.7)	50 (6.1)	
Gender			
Male	73 (52.9)	370 (45.6)	0.11
Female	65 (47.1)	440 (54.4)	
Location			
Ameridian	89 (64.5)	514 (63.5)	0.84
Non Ameridian	49 (35.5)	296 (36.5)	

HBV cleared infection (both anti-HBc/anti-HBs+), chronic inactive/immune controlled HBV infection (anti-HBc + only), previous HBV vaccination (anti-HBs + only), HBV active infection (HBsAg+), individuals susceptible to HBV, and anti-HCV reactivity were found in 12.9, 1.8, 27.2, 0.5, 57.7, 1.2% in Amerindians and 12.1, 2.0, 37.1, 0.3, 55.4, 0.3% in non-Amerindians respectively.

Among HBV immune individuals (*n* = 388), 268 were previously vaccinated (only anti-HBs detected) and 120 had had a previous HBV infection (Table 1). HBV immunity was high in males and individuals aging 21 to 40 years old, but evidence of HBV vaccination (only anti-HBs detected) was more frequent in Amerindian populations, females and those less than 20 years of age.

**Fig. 1:**
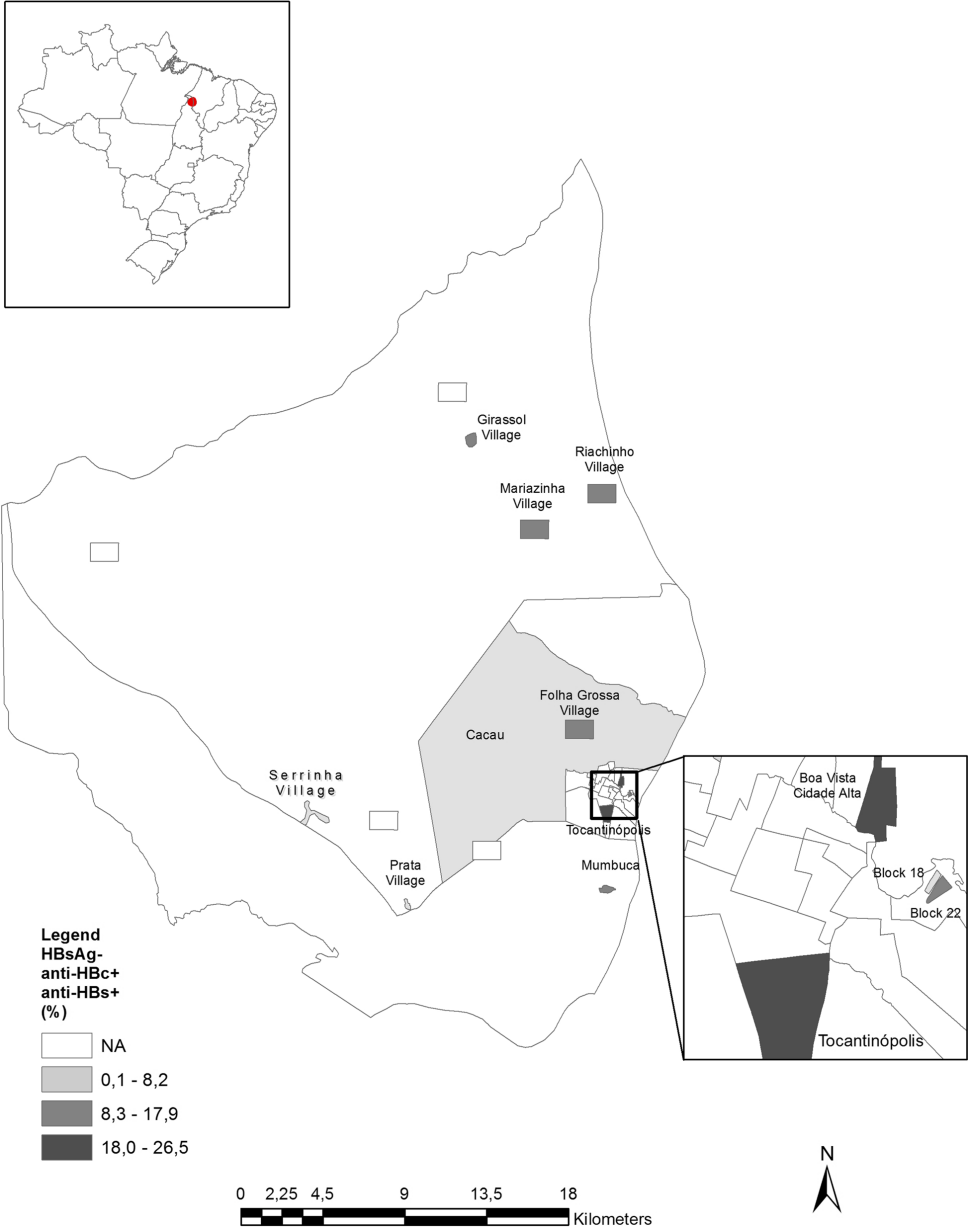
Prevalence of cases of previous HBV infection (HBsAg negative/anti-HBc and anti-HBs positive) according site of recruitment in Eastern Amazon

**Fig. 2:**
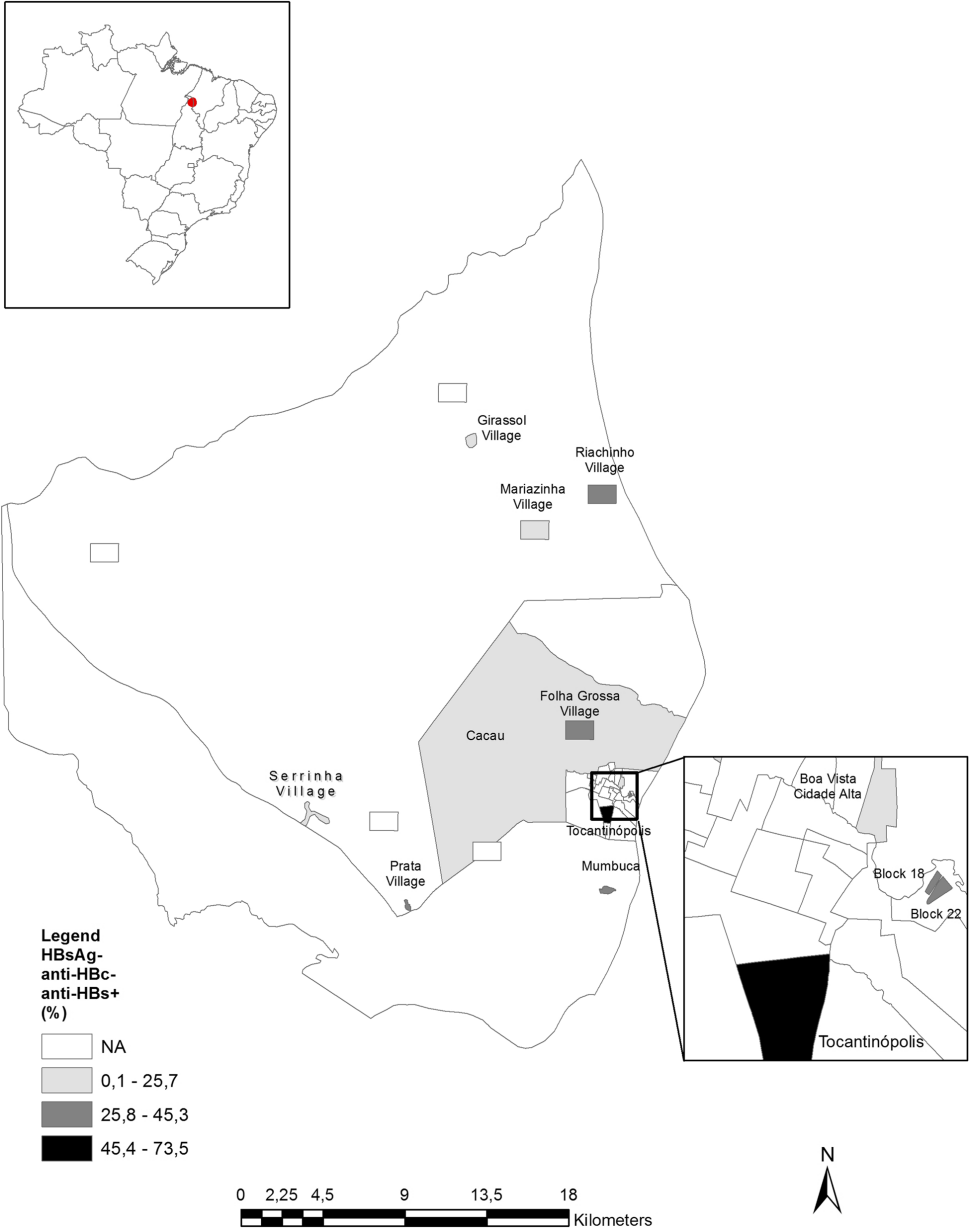
Prevalence of evidence of HBV vaccination (HBsAg and anti-HBc negative/ anti-HBs positive) according site of recruitment in Eastern Amazon

The municipalities with the highest HBV-immune individuals were from Folha Grossa and Mariazinha villages while the lowest number of HBV-immune subjects were seen in Serrinha, Prata, Riachinho and Cacau villages (Figs. 1 and 2).

Anti-Delta was not detected in HBsAg-reactive samples, but it was present in 3 anti-HBc -reactive samples, giving an overall prevalence of 0.3%. Considering these anti-HBc-reactive samples, prevalence of anti-delta was 3.4 and 0% in Amerindians and non-Amerindians respectively. All were male, more than 60 years of age, 2 were from Folha Grossa village and 1 was from Mariazinha village.

Anti-HCV was detected in 8 individuals (7 Amerindian and 1 non-Amerindian), resulting in a prevalence of 1.2 and 0.3% in Amerindians and non-Amerindians respectively and an overall prevalence of 0.8%. High prevalence was observed in males (87.5%) and 50% of individuals aged between 21 to 40 years. HCV RNA was detected in one sample (viral load of 3552 UI/mL).

## Discussion

Parenterally transmitted hepatitis viruses are important threats to public health with Amerindians being potentially more exposed to these infections due to social and cultural habits. Viral hepatitis prevalence has been widely investigated in the Western Amazonian region of Brazil [15–21], but few data are available for the Eastern Amazon region [22, 23], principally in the state of Tocantins. In the present study, new data about HBV, HCV and HDV prevalence were obtained from Eastern Amazon and low prevalence of HBsAg, anti-HCV and anti-delta was observed in these individuals, showing a low risk of transmission of these viruses in this region.

Overall HBsAg prevalence was 0.4%, lower than reported in a previous study from Eastern Amazon (2.88%) [23], and hemodialysis individuals from Tocantins state (4%) [24]. HBsAg prevalence was slightly higher in Amerindians (0.3%) when compared to non-Amerindians (0.1%), but this prevalence is still lower than observed in Amerindians from other localities of the Amazon Region, such as the states of Xingu, Pará and Acre, where prevalence varies from 3.3 to 14.4% [7, 17, 22, 25]. Some authors reported that sociocultural factors such as population density, scarification, tattoos and sexual activity represent important factors in the transmission of HBV [8, 26, 27]. It is possible that the infrequency of these factors among Amerindians from Tocantins may explain their low HBV prevalence compared to other Amerindians from Amazon.

HBV cleared infection was observed in 14.5% of individuals and it was higher among Amerindians and individuals more than 40 years of age. Anti-HBc prevalence varies from 40.7 to 84.7% among Amerindians from Eastern Amazon region [7, 22, 23] and 19.7 to 95.7% among Amerindians from Western Brazilian Amazon [7, 17]. Anti-HBc prevalence was high among older individuals, in concordance with previous studies among non-Amerindians from Amazon region [16, 21, 23].

In the present study, 56.7% of subjects were susceptible to HBV infection and less than 50% were HBV immune, indicating that most individuals are not protected against HBV infection. Although Amerindians are a target group for HBV vaccination, only 27.2% of Amerindians demonstrated isolated anti-HBs reactivity, less than that observed among Amerindians from the Southern (71.0%) and Northern (39.6%) regions of Brazil [22, 28]. It is likely that the low anti-HBs reactivity could be due to poor knowledge about HBV vaccination as previously demonstrated in other groups from the Northern region [29].

Anti-HBs reactivity was irregular among distinct sites of collection where a high prevalence of HBV-immune individuals was found in downtown Tocantinopolis, Folha Grossa and Mariazinha village. This could also be explained by access to health clinics, where high HBV vaccination coverage was found in urban areas. At the time of recruitment (2011), The Brazilian Immunization Program recommended HBV vaccination for all Amerindians and for non-Amerindians newborns and children and adolescents up to 19 years of age. Additionally, the vaccination was also recommended to those at higher risk of acquiring infection such as people who use drug, health professionals, prisoners and other groups [30, 31]. In the present study, high HBV vaccine coverage was found among people aged 0–20 since HBV vaccination was implemented for this demographic.

Overall an anti-HCV prevalence of 0.8% was observed in individuals from Tocantinópolis city, lower than that observed in studies of the general population from the Eastern Amazon Region (2.2 to 5.76%) [23, 32, 33] or hemodialysis individuals from Tocantins state (13%) [24]. However, anti-HCV prevalence was high in Amerindians (1.2%) compared to non-Amerindians (0.3%) in concordance with a previous study from the Northern region (1.4%) [14].

Some studies have demonstrated that sharing cutting instruments among relatives and/or neighbors is a common habit in individuals from the Amazon region [34, 35]. These cultural habits could increase the risk of transmission of parenterally acquired viruses, such as, HCV. In addition, low knowledge about hepatitis transmission [29] and low access to treatment service could contribute to this high prevalence.

Overall anti-delta prevalence was 0.3% and all of these individuals were Amerindians, giving a prevalence of 3.4% in this group. According to the Brazilian Health Ministry, 10 confirmed cases of hepatitis D were found in Tocantins from 1999 to 2016 giving a prevalence of 0.3% in Brazil [6]. Previous studies did not find anti-delta antibodies in Amerindians from the Eastern Amazon Region, [22, 25] while an anti-delta prevalence of 2.9 to 7.3% was observed in the Western Amazon Region [15, 17, 21].

Of the residents in the northern Area of Tocantins state, approximately 4.4% are reported to be Amerindian, with 81% of the population living in urban areas [36]. In the present study, 60% of participants were Amerindian and 64% reported living rurally/in villages. The main purpose of this study is to provide new insights regarding hepatitis prevalence in Amerindians living in rural areas, so a high proportion of this group was included in the study. A major limitation of the study was the type of sampling. Consecutive sampling was used due to impossibility to conduct probability sampling in this study and since the method is very cost- and time-effective. However, it is not possible to generalize the results of the survey to the entire population and there is the possibility of under- or over-representation of the population.

## Conclusions

Overall low prevalence of HBV and HCV infection was found in the population studied, but higher HBV and HCV prevalence was observed among Amerindians compared to the urban population, likely due to inadequate access to prevention or treatment services due to rural or remote location. Moreover, anti-delta antibodies were found only among Amerindians from the Eastern Amazon, suggesting a risk for infection in this population. Of note is that nearly half of Amerindians had no anti-HBs, indicating the need for HBV vaccination campaigns in this population. These findings should serve as an important baseline for future primary prevention and therapeutic intervention strategies.

## Abbreviations

Anti-HBc IgM: Antibodies directed against the core antigen IgM
Anti-HBc total: Antibodies directed against the core antigen
Anti-HBe: Antibodies against HBeAg
Anti-HBs: Antibodies directed against hepatitis B surface antigen
Anti-HCV: Antibodies directed against hepatitis C virus
Anti-HDV: Antibodies directed against hepatitis D virus
ECLIA: Electrochemiluminescence
ELISA: Enzyme immunoassay
HBeAg: HBV “e” antigen
HBsAg: Surface antigen of the hepatitis B virus
HBV: Hepatitis B virus
HCV: Hepatitis C virus
HDV: Hepatitis D virus
IBGE: Brazilian Institute of Geography and Statistics
